# A Flexible-Integrated Multimodal Hydrogel-Based Sensing Patch

**DOI:** 10.1007/s40820-025-01656-w

**Published:** 2025-02-21

**Authors:** Peng Wang, Guoqing Wang, Guifen Sun, Chenchen Bao, Yang Li, Chuizhou Meng, Zhao Yao

**Affiliations:** 1 School of Information Science and Engineering, University of Jinan, Jinan, 250022 People’s Republic of China; 2 School of Mechanical Engineering, University of Jinan, Jinan, 250022 People’s Republic of China; 3 State Key Laboratory for Reliability and Intelligence of Electrical Equipment, Engineering Research Center of Ministry of Education for Intelligent Rehabilitation Device and Detection Technology, Hebei Key Laboratory of Smart Sensing and Human-Robot Interaction, School of Mechanical Engineering, Hebei University of Technology, Tianjin, 300401 People’s Republic of China; 4 School of Integrated Circuits, Shandong University, Jinan, 250101 People’s Republic of China; 5 College of Electronics and Information, Qingdao University, Qingdao, 266071 People’s Republic of China; 6 State Key Laboratory of Integrated Chips and Systems, Fudan University, Shanghai, 200433 People’s Republic of China

**Keywords:** Multimodal sensing, Proximity sensor, Pressure sensor, Temperature sensor, Electrospun nanofibers

## Abstract

Sleep monitoring is an important part of health management because sleep quality is crucial for restoration of human health. However, current commercial products of polysomnography are cumbersome with connecting wires and state-of-the-art flexible sensors are still interferential for being attached to the body. Herein, we develop a flexible-integrated multimodal sensing patch based on hydrogel and its application in unconstraint sleep monitoring. The patch comprises a bottom hydrogel-based dual-mode pressure–temperature sensing layer and a top electrospun nanofiber-based non-contact detection layer as one integrated device. The hydrogel as core substrate exhibits strong toughness and water retention, and the multimodal sensing of temperature, pressure, and non-contact proximity is realized based on different sensing mechanisms with no crosstalk interference. The multimodal sensing function is verified in a simulated real-world scenario by a robotic hand grasping objects to validate its practicability. Multiple multimodal sensing patches integrated on different locations of a pillow are assembled for intelligent sleep monitoring. Versatile human–pillow interaction information as well as their evolution over time are acquired and analyzed by a one-dimensional convolutional neural network. Track of head movement and recognition of bad patterns that may lead to poor sleep are achieved, which provides a promising approach for sleep monitoring.

**Supplementary Information:**

The online version contains supplementary material available at 10.1007/s40820-025-01656-w.

## Highlights

- A flexible multimodal proximity–pressure–temperature sensing patch with a simple structure was developed.
- Thanks to structural design and material synthesis, the sensor has an outstanding temperature sensitivity of 0.5 °C^−1^ with good linearity, a high pressure sensitivity of 30.6 kPa^−1^ and a non-contact sensing range is 2 m.
- After the multimodal sensing patches are integrated at different locations of the pillow, the sleeping conditions can be monitored comfortably.

**Supplementary Information:**

The online version contains supplementary material available at 10.1007/s40820-025-01656-w.

## Graphical abstract

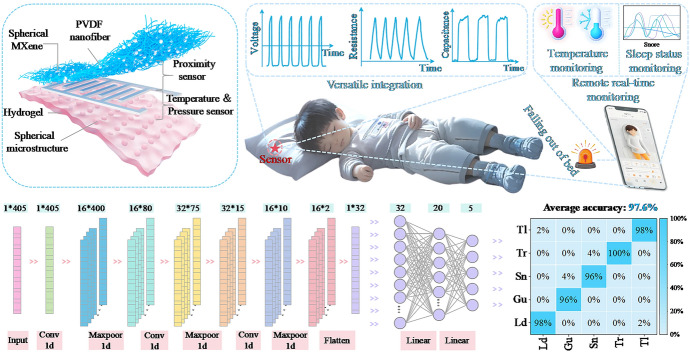

## Introduction

With the fast development of economic levels and living standards, people pay increasing attention to health status. Among various aspects of health management, sleep monitoring is an important one, because around one-third of a person's life is spent in sleep and the quality of sleep is crucial for the restoration of human health. Even though traditional sleep monitoring methods, such as polysomnography (PSG) and portable devices, can provide detailed sleep data, they must be conducted in a fixed testing environment where complex wires are connected to bulky complex equipment, severely limiting their popularized application and popularization in daily life. In contrast, wearable sensors, due to their capabilities in physiological signal detection and posture recognition with small formation and flexible properties, can be utilized as an alternative way for sleep monitoring [1, 2]. But, they still need to be tightly worn on multiple parts of the human body with connecting wires or transmission antennae to acquire the sensing information, which would inevitably interfere with the quiet sleeping experience. Therefore, it is highly desired to develop an effective approach to monitoring sleep in a comfortable unrestricted way. And, to meet the need for the sleeping monitor, multifunctional-integrated sensors have also been developed [3–5]. However, the existing multifunctional-integrated sensors have complex structures (containing more than four layers), and long-term use can lead to interface separation, performance degradation, and reduced wearing comfort. Thus, a multifunctional sensor with little layer structure is needed.

In recent years, wearable sensors have made significant progress in various applications of disease perception, posture detection, and physiological signal monitoring [6–9]. Among available polymer materials as flexible substrates, hydrogels, composed of interlinked polymer networks containing a large content of water, have unique properties [10–12] such as self-adhesive, adjustable conductivity, and similar-to-biological-tissue modulus, and good biocompatibility, making them a promising substrate candidate to construct flexible sensors to be worn on the human body in a tight and conformal way [13–18]. However, the practical application of hydrogel sensors still faces main challenges. Due to the high-water content, hydrogels show limited mechanical properties with low strength and toughness, not suitable in some severe flexible conditions [19–24]. Additionally, the water content of hydrogels will significantly decrease after a long time, leading to poor durability [25–28]. The loss of water due to evaporation not only affects the mechanical strength but also reduces the sensing capability. Moreover, most hydrogel sensors only offer single sensing functions, such as pressure [29–32] or temperature [33–36], which fails to meet the diverse demand of simultaneously monitoring multiple human parameters in complex environments [37–40]. Furthermore, most acquired sensing signals such as pressure and temperature are the biological information on human skin. To extend the functionality of flexible sensors, interaction information arising from non-contact behavior within a short distance range between approaching objects and the human body is also necessary, so proximity sensors are needed [41–45]. Therefore, developing hydrogel sensors with strong mechanical toughness and long-lasting water retention as well as multimodal sensing capability is greatly significant to push the personal health monitoring technique to a high level.

In this work, we report the development of a flexible-integrated multimodal proximity–pressure–temperature sensing patch (M-PPT) based on strong toughness and water retention hydrogel and its application in unconstraint sleep monitoring. The sensing patch consists of a hydrogel-based bimodal pressure–temperature sensing layer on the bottom and an electrostatically spun nanofiber-based non-contact detection layer on the top. To enhance the pressure sensing sensitivity of the hydrogel, we introduced a microspherical structure design on the surface of the hydrogel, which enables the sensor to detect small pressure changes more sensitively and is suitable for complex physiological signal monitoring scenarios. In addition, at the bottom of the electrostatically spun nanofibers, a fork-finger-like electrode layer was prepared by magnetron sputtering. It can be used as an electrode layer for proximity–pressure–temperature sensing at the same time. The hydrogel as the core substrate material exhibits excellent mechanical properties with no decline in strength toughness and deformation recovery after cycling and outstanding water retention with no weight loss after 8 days. The internal structure effectively locks the moisture, preventing water evaporation, which ensures the hydrogel sensor maintains its original sensing performance during extended use. Regarding the multimodal sensing capability, the temperature sensing based on the conducting polymer of poly(3,4-ethylenedioxythiophene)/poly(styrenesulfonate) (PEDOT:PSS) yields a high sensitivity of 0.5 °C^−1^ with a good linear relationship, favorable in body temperature monitoring; the pressure sensing based on the iontronic supercapacitive sensing mechanism exhibits a high sensitivity of 30.6 kPa^−1^ at low pressure range of 1 kPa and 26.3 kPa^−1^ at high pressure range of 40 kPa with a fast response time of 5.6 ms, beneficial in head touch detection; the non-contact sensing based on the MXene-doped triboelectric polyvinylidene fluoride (PVDF) nanofibers has an ultra-wide non-contact detection range of over 2 m with stabile durability, facilitating in proximity interaction sensing. To validate the practicability of M-PPT, the multimodal sensing function is verified in a simulated real-world scenario by robotic hand grasping objects. In the end, multiple multimodal sensing patches seamlessly integrated on different locations of a pillow for intelligent sleep monitoring are achieved, where versatile human–pillow interaction information including temperature, pressure, and proximity signals as well as their evolution with the head movement are simultaneously acquired in a real-time way and a one-dimensional convolutional neural network (1D CNN) is applied to analyze the multidimensional sensing data for track of head movement and recognition of bad patterns that may lead to poor sleep. In this way, users can monitor their sleep quality in a comfortable unrestricted way and have personalized sleep management to prevent potential health risks.

## Experimental Section

### Materials

Polyvinylidene fluoride (PVDF)and 1-ethyl-3-methylimidazolium bis(trifluoromethanesulfonimide) salt ([EMIM][TFSI]) were purchased from Shanghai Aladdin Biochemical Technology Co. Acrylamide (AM, Analytical Reagent, hereinafter referred to as AR, 99.0%), titanium aluminum carbide powder (Ti_3_AlC_2_), PEDOT:PSS (1.5% solids in water), N,N'-methylenebisacrylamide (MBA, AR), ammonium persulfate (APS, AR, 98.5%), lithium fluoride (lixivium fluoride), and Gly (AR, 99%) were purchased from Macklin Biochemical Co. Ltd (Shanghai, China).

### Preparation of Sensor Patches

#### Fabrication of MXene (Ti_3_C_2_T_x_) Nanosheets

(1) Add 20 mL of 75% concentrated hydrochloric acid and 1.7 g of lithium fluoride to 10 mL of deionized water, and stir with a magnetic stirrer at 1200 r min^−1^ for 10 min until the lithium fluoride is completely dissolved in the hydrochloric acid solution. (2) Stir the mixed solution at 600 r min^−1^ with a magnetic stirrer, and slowly add 1.3 g of titanium carbide aluminum into the etching solution. Then heat in a water bath at 40 °C and stir at 1000 r min^−1^ for 24 h to remove aluminum and obtain the MXene etching solution. (3) Centrifuge the etching solution at 8000 r min^−1^ for 5 min to obtain a multilayer MXene precipitate. After mixing with deionized water, place it in a freeze dryer for 24 h to obtain multilayer MXene powder.

#### Fabrication of Spherical Microstructure Molds

(1) Use 3ds Max 2024 software to design a rectangular prism with dimensions of 1.8 cm × 1.5 cm × 0.5 cm. Many spheres with a radius of 250 μm are arranged on the top surface of the rectangular prism. (2) Use UV-sensitive resin material for 3D printing to create the mold. Clean the surface of the mold slowly with anhydrous ethanol to remove impurities, then cure it with UV light. (3) Prepare PDMS by thoroughly mixing the curing agent with the base monomer (Dow Corning Sylgard 184, with a weight ratio of the base to the crosslinker of 10:1). (4) Pour the PDMS mixture into the printed mold, de-gas under vacuum at room temperature for 20 min to remove bubbles, then cure completely at 80 °C for 2 h, and peel off to obtain a mold with recessed spherical microstructures.

#### Preparation of M-PPT Organic Hydrogels Containing Spheroid Microstructures

(1) Add 4.2 g of AM powder and 0.6 g of PVA pellets to 8 mL of deionized water, and stir with a magnetic stirrer at 1000 r min^−1^ at 85 °C for 3 h. (2) Add 1 mL of 5% MXene aqueous solution and 1 mL of PEDOT:PSS solution to the above solution, and stir with a magnetic stirrer at 600 r min^−1^ at room temperature for 2 h. (3) Slowly add 1.2 mL of [EMIM][TFSI] to the solution and stir with a magnetic stirrer at 600 r min^−1^ for 30 min. (4) Sequentially add 0.42 mL of 20% APS initiator solution and 0.23 mL of 2% MBA solution to the mixture. (5) Place the prepared spherical microstructure mold in a glass petri dish (treated with a Plasma Cleaner). (6) After thoroughly mixing the solution, pour the resulting mixture onto the spherical microstructure mold. (7) Degas the solution, then place the petri dish in a 60 °C oven for 20 min to obtain an M-PPT hydrogel containing spherical microstructures. (8) Finally, soak the obtained M-PPT hydrogel in a Water/Gly (mass ratio: 1:1) mixed solution for 10 min to obtain the final M-PPT organic hydrogel.

#### Preparation of PVDF/Ti_3_C_2_T_x_ Nanofibers for Triboelectric Proximity Sensing Layer

(1) Dissolve 2 g of PVDF powder in 8 mL of DMF solvent. (2) Heat the solution in a water bath at 80 °C and stir at 800 r min^−1^ for 3 h to ensure complete dissolution. (3) Add 0.05 g of Ti_3_C_2_T_x_ powder to the solution and stir with a magnetic stirrer at 600 r min^−1^ for 1 h to ensure the Ti_3_C_2_T_x_ powder is evenly dispersed. (4) Use a 10 mL disposable syringe to take 8 ml of the mixed solution for electrospinning. Set the extrusion speed to 0.6 mm min^−1^, the collection distance to 80 mm, the spinning time to 2 h, and the spinning voltage to 18 kV. (5) Dry the electrospun membrane in a vacuum at 40 °C for 12 h to remove surface moisture.

#### Preparation of Ag/PVDF/Ti_3_C_2_T_x_ Nanofiber Fork Finger Electrodes

The patterned interdigitated electrode mask is placed on the PVDF/Ti_3_C_2_T_x_ film and secured to the magnetron sputtering substrate using clamps. Commercial silver target material is sputtered using direct current electromagnetic control with an oxygen flow rate of 20, a pressure of 2 Pa, a power of 80 W, and a sputtering time of 15 min. Finally, an Ag/PVDF/Ti_3_C_2_T_x_ nanofiber membrane with patterned interdigitated Ag electrodes is obtained.

## Results and Discussion

### Design Concept and Synthesis Strategy of the M-PPT

**Fig. 1:**
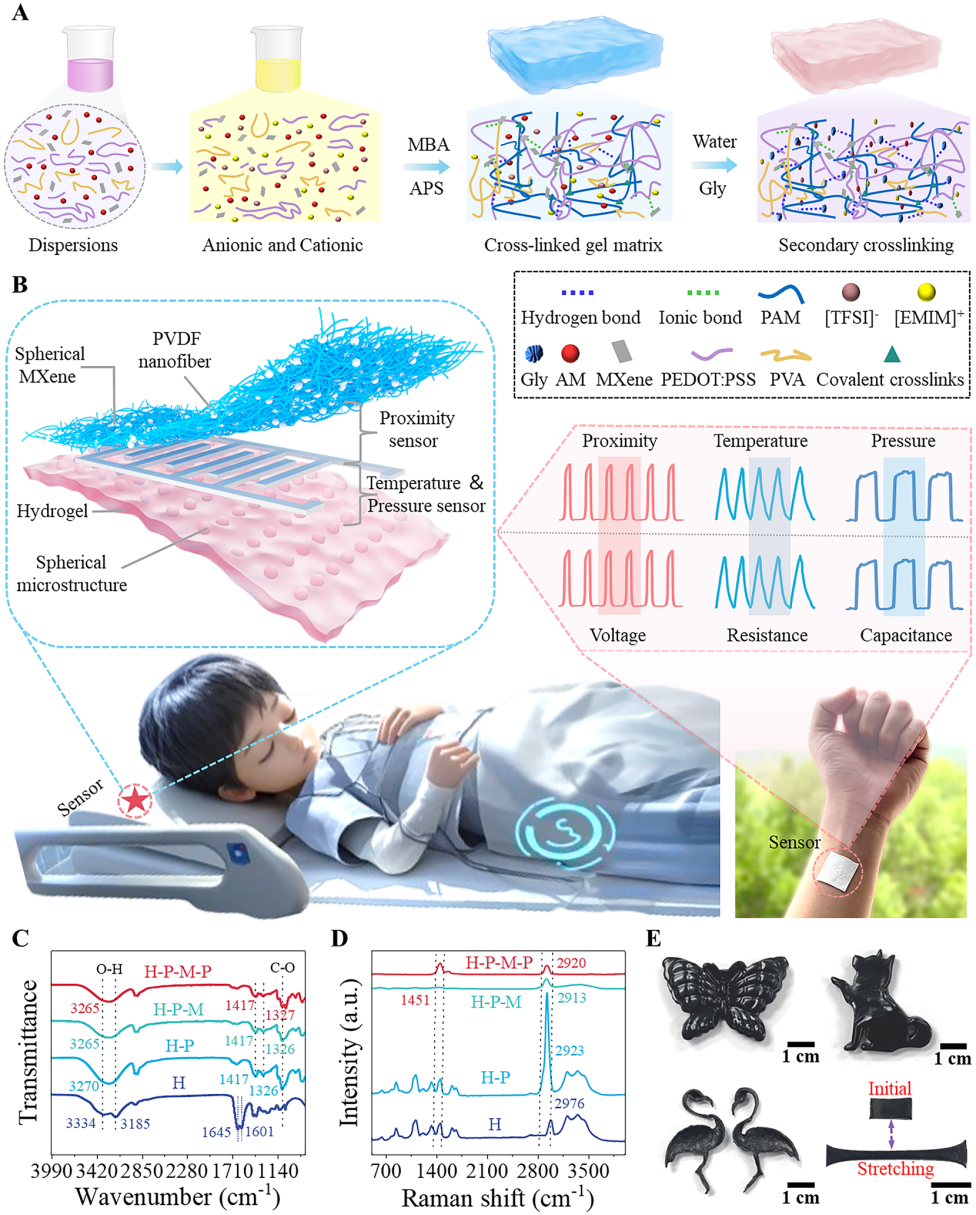
Design concept and synthesis strategy of hydrogel-based M-PPT for sleep monitoring. **A** Preparation flow chart of the hydrogel with ionic cross-linking structure. **B** Schematic diagram illustrating the structure, functionality, and application of the M-PPT. **C** FTIR spectral analysis and **D** Raman spectral analysis of the hydrogel. **E** Photographs showing the hydrogel molded into various shapes as well as its superior stretchability

Hydrogel, as an intrinsic flexible material, is ideal for sensor design due to its unique physical and chemical properties. Figure 1A shows the preparation process of the dual-mode pressure–temperature sensor based on hydrogel with its ionic cross-linking structure. During the preparation process **(**Fig. S1**)**, the successful initiation of the structural ionic cross-linking is crucial, which not only enhances its internal mechanical strength but also provides sensitivity to external pressure and temperature stimuli. Figure 1B illustrates the overall structure, functionality, and application of the proposed multimodal proximity–pressure–temperature sensing path (M-PPT). The photograph showing the physical appearance of the electronic path can be found in Fig. S7. Regarding the internal device structure, the hydrogel-based dual-mode pressure–temperature sensing layer is laid at the bottom serving as the biocompatible sticky layer attaching to human skin, and a triboelectric proximity sensing layer with interdigitated silver electrodes is integrated at the top. Toward practical human health monitoring, the M-PPTs can be seamlessly placed at multiple positions on a commercial pillow, which can be used to identify the sleeper’s head position in a continuous, real-time way through intelligent recognition of the multimodal sensing information of temperature, pressure, and proximity distribution. This kind of versatile data is significant for in-depth sleep study in an unrestricted manner. The acquired diverse signals representing different physiological parameters show characteristic waveforms during different sleep stages, providing scientific evidence for sleep quality assessment and sleep disorder diagnosis.

Polyacrylamide (PAM) is used in hydrogel matrices due to its 3D mesh structure, water solubility, non-toxicity, and stability, with high tensile properties but low mechanical strength. MXene nanosheets, on the other hand, have been introduced to build stable 3D conductive networks with enhanced mechanical properties due to their excellent water dispersibility, chemical stability, and electrical conductivity. PEDOT:PSS is selected for the positive–negative charges of its positive–negative polymer chains. PEDOT:PSS is used due to its positively and negatively charged polymer chains, which can form electrostatic interactions with the charged components in the hydrogel, and the sulfonic acid groups of the PSS chains can be combined with the hydrogel network through hydrogen bonding to form a three-dimensional conductive network. Polyvinyl alcohol (PVA) is selected because it can form strong hydrogen bonds with other components (MXene and PEDOT:PSS) through the hydroxyl groups in its chains, thus enhancing the mechanical properties of the hydrogel. To verify the chemical composition and structure of the hydrogel, Fourier transform infrared spectroscopy (FTIR) and Raman spectroscopy analysis are employed. FTIR spectroscopy displays characteristic absorption peaks of various functional groups, which confirms the specific chemical bonds and molecular structures in the hydrogel matrix **(**Fig. 1C**)**. The characteristic peaks at 3185 cm^−1^ belong to the tensile vibration of N–H in H hydrogel (Ordinary AM hydrogel). The absorption peak at 1417 cm^−1^ is attributed to the stretching vibration of the carbonyl group, while the peak at 1326 cm^−1^ is due to the bending vibration of the amide group's N–H. When hydrogen bonds form between molecules, the -OH stretching peak undergoes vibration [46]. Compared to H-P hydrogel (Hydrogel after the addition of PVA), the -OH characteristic peak of H-P-M-P hydrogel (Hydrogel after the addition of PVA-MXene-PEDOT:PSS) shifts significantly from 3270 to 3265 cm^−1^, indicating the formation of hydrogen bonds between H-P-M-P. Raman spectroscopy provides further information on the molecular structure of the hydrogel **(**Fig. 1D**)**, where a peak at 1451 cm^−1^ is observed in H-P-M-P hydrogel due to the addition of PEDOT:PSS. From a macroscopic view, the hydrogel exhibits superior flexibility as shown in Fig. 1E, where various forms such as butterflies, pet dogs, and flamingos can be shaped, guaranteeing its qualification as a flexible substrate for wearable sensors. The hydrogel also shows excellent stretchability to be used in severe flexible conditions **(**Fig. S2**)**. To further validate the flexibility of the sensor patch, we subjected the complete sensor piece to bending and twisting tests **(**Fig. S3**)**, and the sensing patch maintains good flexibility and can adapt to complex working environments such as bending and twisting.

By adding glycerin, the hydrogel exhibits excellent water retention capability, with almost no shrinkage in appearance **(**Fig. S4**)** and no water content loss in weight **(**Fig. S5**)** over a long time of 8 days. This indicates that the hydrogel is capable of maintaining moisture over a long period, ensuring superior stability and reliability for flexible sensors. To verify the multi-water structure within the hydrogel, we subjected hydrogel samples to freeze-drying treatment. By freezing the water content in the samples into a solid state and then sublimating it under low temperature and low pressure conditions, significant changes in the internal structure of the hydrogel occurred due to the removal of water, resulting in the formation of a porous structure (Fig. S6). This porous structure directly reflects the multi-water network within the hydrogel.

### Temperature Sensing Performance of the M-PPT

**Fig. 2:**
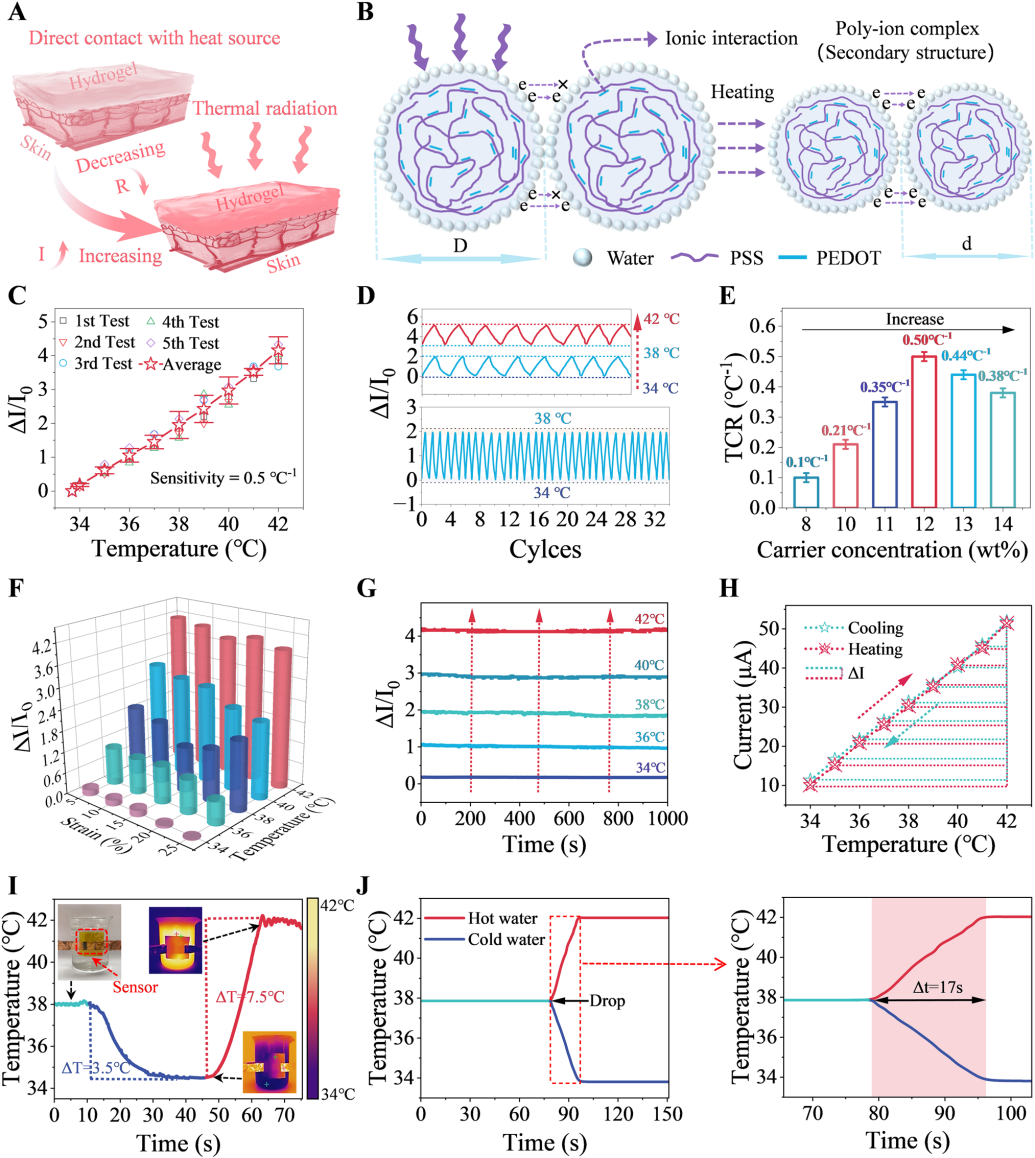
Mechanism and performance of the temperature sensing mode. **A** Schematic diagram illustrating the working principle of the heat transfer of hydrogel from the skin and **B** the moisture release of PEDOT and PSS molecules. **C** Sensitivity and **D** cyclic performance of the sensor. **E** Sensitivity of the sensor at different carrier concentrations. **F** Performance changes of the sensor under different stretching and temperature conditions. **G** Stability of the sensor at different temperatures. **H** Linear fitting of the sensing signal of the sensor during heating and cooling cycles. **I** Detection of water with different temperatures and **J** corresponding response recovery time

Figure 2 provides a detailed demonstration of the working principle and several key performance aspects of the hydrogel-based temperature sensor. First, Fig. 2A shows a schematic of the hydrogel temperature sensor's operation, illustrating how the sensor resistance decreases and current increases when the external temperature changes upon contact. Figure 2B provides a detailed schematic of the working principle of PEDOT:PSS molecules. PEDOT:PSS is a conductive polymer material commonly used in flexible electronic devices. It has a unique core–shell structure, where the conductive PEDOT:PSS core is surrounded by an insulating PSS shell. When the temperature rises, water molecules in the hydrophilic PSS are released into the external environment, causing the PSS shell to shrink. This reduces the distance between adjacent PEDOT cores, enhancing electron hopping between them. Consequently, the resistance of PEDOT:PSS decreases with increasing temperature [47–49]. In the temperature sensing system, carriers in the hydrogel are transported to form a stable ion flow. Ion transport is a thermally activated process, i.e., the rate of ion transport increases as the temperature of the hydrogel increases. The faster the rate of ion transport, the lower the resistance of the temperature sensing hydrogel. In addition, an increase in temperature triggers microscopic phase transitions within the hydrogel material that can further optimize the ion transport path. The increase in temperature also generates a temperature difference that further drives carrier transport. Figure 2C shows the sensitivity of the temperature sensor. The average data from five experimental tests were used to plot the sensitivity curve of the sensor, which exhibits a maximum sensitivity of 0.5 °C^−1^. Δ*I*/*I*_0_ is calculated by rationing the difference in the current before and after the temperature change (Δ*I* = *I*-*I*_0_) to the initial current value, Δ*I* represents the change in current measured by the sensor at different temperatures, *I*_0_ represents the current value at the initial state of the experiment, and *I* represents the current value after the temperature change. To verify the stability and reliability of the sensor under repeated temperature changes, cyclic temperature variation tests were conducted. The results show that the sensor demonstrates excellent cyclic stability within two temperature ranges. Figure 2D presents the sensor's cyclic performance at different temperatures of 34–38 and 38–42 °C (To highlight the performance of the sensor, the narrow range of experimental temperatures is selected by us). The sensor maintains consistent performance over prolonged use without significant degradation. To determine the effect of different carrier concentrations on sensor sensitivity, comparative analysis of experimental data shows that the sensor's sensitivity is highest at a carrier concentration of 12 wt% (as shown in Fig. 2E**)**. Due to the excellent flexibility and stretchability of the hydrogel material, studying its temperature response under different stretching conditions is crucial. Figure 2F shows the performance variation of the sensor under different stretching and temperature conditions. The results indicate that the sensor maintains high sensitivity and stability under various stretching states. Additionally, the sensor can maintain stable signal output under long-term constant temperature conditions, which is essential for continuous monitoring in practical applications.

Figure 2G shows the experimental results of the sensor maintaining stable performance at temperatures of 34, 36, 38, 40, and 42 °C. There is no significant drift or noise, ensuring the reliability and accuracy of the temperature data. In Fig. 2H, the signal change of the sensor under heating and cooling cycles is displayed. The experimental results show that the sensor's signal change has a good linear relationship, indicating that it can provide stable and predictable electrical signal output during temperature changes. This linear characteristic is beneficial for the calibration and data processing of temperature sensors, simplifying data conversion and analysis during temperature measurement. To verify the sensor's performance in practical applications, it was attached to the outside of a beaker to test water at different temperatures (as shown in Fig. 2I). The sensor accurately detected the water temperature changes. Figure 2J shows the recovery time of the sensor from temperature changes to stabilization between 38 and 42 °C, which is 17 s. The rapid recovery time means that the sensor can quickly return to its initial state (Fast temperature recovery mechanism is given in Note S3), improving the real-time and accuracy of temperature measurements, meeting the needs of real-time monitoring. To comprehensively evaluate the sensor’s performances, we compared the sensing time and sensitivity with similar reported works, as shown in Fig. S8. It can be found that the sensitivity and response time of the sensing device prepared by us are superior to the reported works, which benefit from the cooperative sensing mechanism of PEDOT:PSS and MXene.

### Pressure Sensing Performance of the M-PPT

**Fig. 3:**
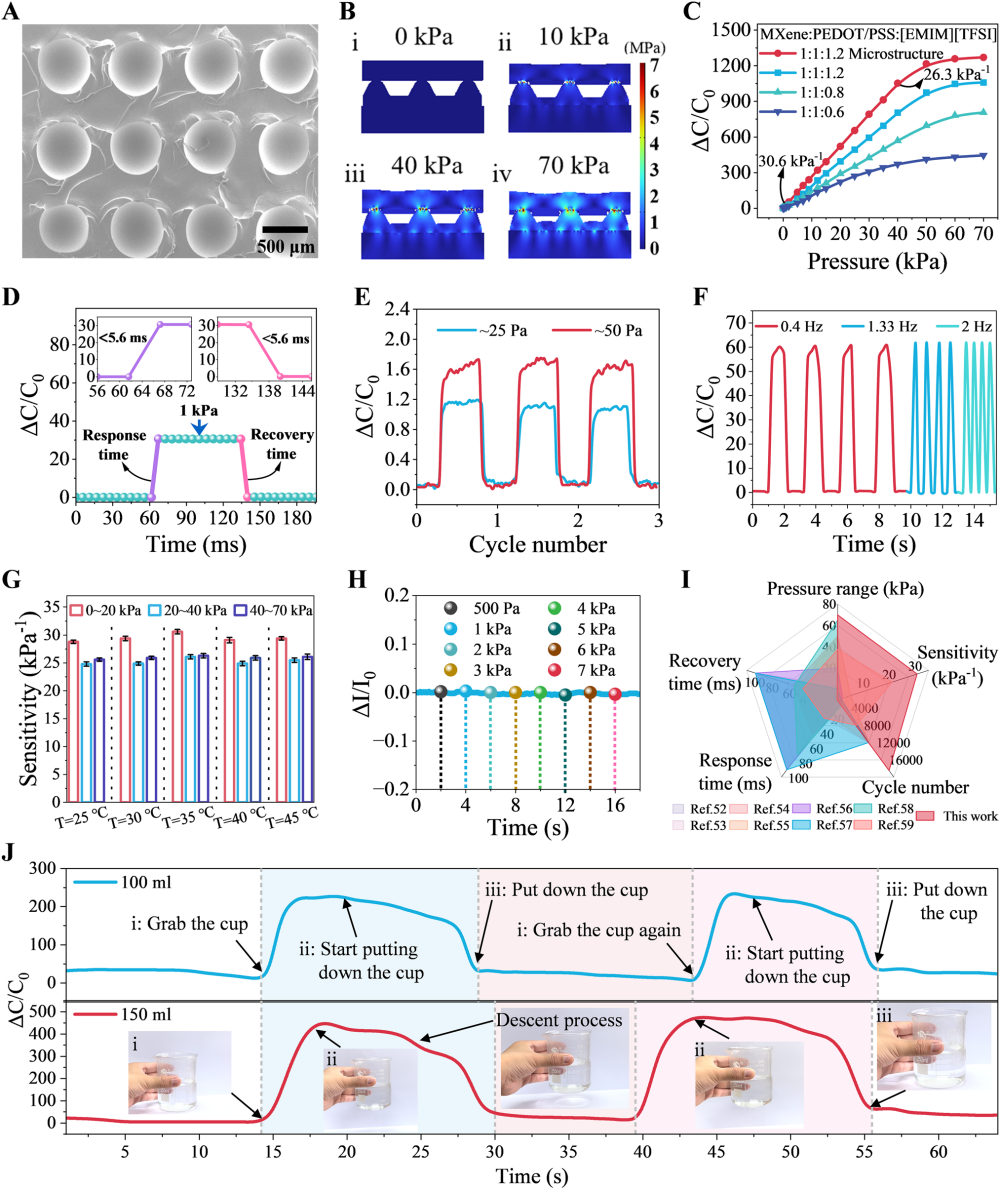
Mechanism and performance of the pressure sensing mode. **A** SEM image of the surface microstructures on the hydrogel layer. **B** COMSOL simulation of the pressure sensing process. **C** Sensitivity curves of the sensors with different compositions. **D** Response and recovery time, **E** minimum detection limit, and **F** dynamic response signals under different frequencies of the sensor. **G** Temperature influence on the pressure sensing mode. **H** Pressure influence on the temperature sensing mode. **I** Performance comparison of the sensor with previously reported works. **J** Detection of grasping cups with different water contents by hand

Figure 3 showcases the performance characteristics and working mechanism of the hydrogel-based pressure sensor, validated through a series of experiments and simulations, demonstrating its excellent performance and wide application potential. To enhance the sensor's sensitivity and response speed, a spherical microstructure was fabricated on the hydrogel surface using a pattern transfer method. These microstructures reduce the initial contact area, playing a crucial role during sensing. The surface spherical microstructure of the hydrogel pressure sensing layer was characterized using scanning electron microscopy (SEM) (as shown in Fig. S9), revealing the ordered arrangement of the spheres **(**Fig. 3A**)**. Next, the behavior of the sensor under applied pressure was simulated using COMSOL software **(**Fig. 3B**)**, allowing observation of internal structural changes and stress distribution when pressure is applied to the hydrogel surface. Due to its ionic supercapacitor sensing mechanism and spherical microstructure, the proposed electronic skin exhibits excellent sensing performance. The working principle of the fabricated pressure sensor is illustrated in Fig. S10. Figure 3C presents the relative capacitance change curves (sensitivity curves) of sensors with different ionic contents. Sensitivity is a key parameter for pressure sensing, defined as $S=\delta (\Delta C /{C}_{0})/\delta P$, where $\Delta C$ is the change in relative capacitance [50], ${C}_{0}$ is the initial capacitance without pressure, and $P$ is the applied pressure. Among the four different sensors studied, the one with spherical microstructures and an MXene:PEDOT:PSS:[EMIM][TFSI] ratio of 1:1:1.2 exhibited the highest pressure sensitivity, with a sensitivity of 30.6 kPa^−1^ at a low pressure of 1 kPa and 26.3 kPa^−1^ at a high pressure of 40 kPa. Figure 3D displays the dynamic response speed of the hydrogel-based pressure sensor under pressure changes. The results show that the sensor responds quickly to pressure changes and rapidly returns to its initial state after pressure release, with a response and recovery time of less than 5.6 ms. Figure 3E demonstrates the sensor's minimum detection limit, capable of detecting small pressures of 25 and 50 Pa. To verify the sensor's performance under dynamic loads, pressure application tests at different frequencies (0.4, 1.33, and 2 Hz) were conducted **(**Fig. 3F**)**. The sensor consistently outputted stable signals at different frequencies, indicating good frequency response characteristics. Additionally, the cyclic stability of the hydrogel-based pressure sensor was tested (as shown in Fig. S11). After approximately 16,500 cycles, the sensor still maintained excellent stability. Since environmental temperature changes can affect sensor performance, it is crucial to understand how temperature impacts pressure sensing performance. Figure 3G investigates the sensor's pressure performance at different temperatures. The results show that the hydrogel-based pressure sensor maintains high sensitivity and stability within the temperature range of 25–45 °C, indicating good temperature stability (as shown in Fig. S12). Because the pressure and temperature sensors are integrated into the same device, studying the effect of pressure on temperature sensing performance is also essential. Figure 3H shows the impact of different pressures (500 Pa-7 kPa) on temperature sensing performance. The results indicate that the sensor's temperature response remains stable under varying pressure conditions, demonstrating minimal interference between pressure and temperature measurements, ensuring reliable simultaneous measurements. A comparison of the hydrogel-based pressure sensor's performance with other reported works is provided in Fig. 3I. The designed sensor demonstrates significant advantages in sensitivity, response and recovery time, working range, and stability [51–58]. To validate the sensor's feasibility and reliability in practical applications, it was attached to cups containing different amounts of water (100 and 150 mL). The sensor accurately detected pressure changes when the cups were held by hand **(**Fig. 3J**)**. Additionally, the hydrogel-based pressure sensor precisely sensed pressure changes under different wrist bending angles (30°, 60°, and 90°) (as shown in Fig. S13). The sensor also successfully achieved pulse detection, capturing subtle pulse fluctuations (Fig. S14), and providing accurate physiological data.

### Proximity Sensing Performance of the M-PPT

**Fig. 4:**
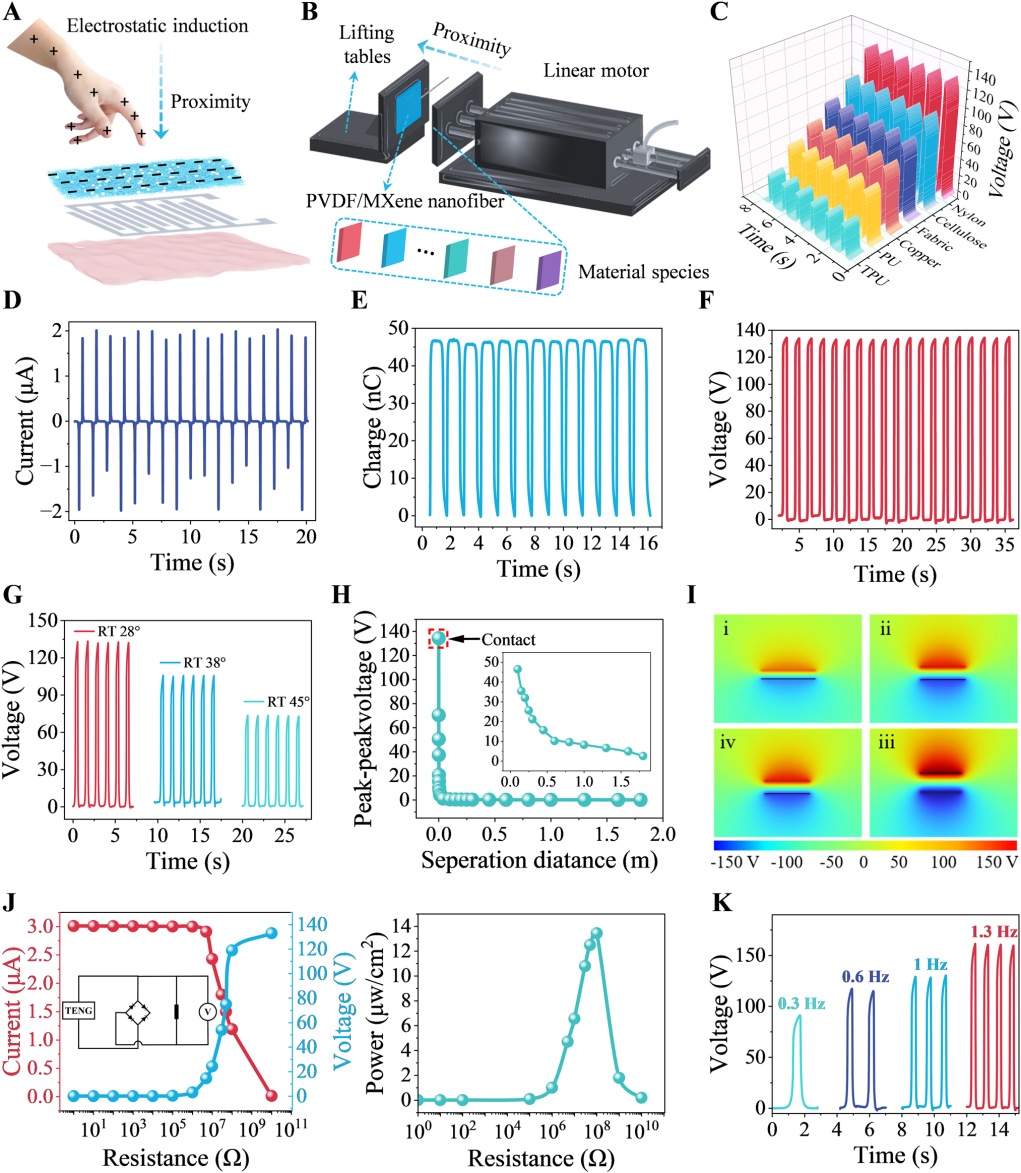
Mechanism and performance of the proximity sensing mode. **A** Schematic diagram illustrating the working principle of proximity sensing based on triboelectric effect in a vertical mode. **B** Schematic diagram illustrating the collision and friction testing setup between the proximity sensor and various counterpart materials. **C** Response voltages between the proximity sensor and different counterpart materials. **D** Current, **E** charge, and **F** voltage between the proximity sensor and nylon material. **G** Temperatures influence the voltage of the proximity sensor. **H** Voltage decreases with increasing separation distance. **I** COMSOL simulation for the proximity sensor. **J** Peak power under different loads. **K** Voltage changes at different frequencies

Our proximity sensor primarily employs the triboelectric principle, with a schematic diagram of the vertical mode shown in Fig. 4A. In this mode, an electric signal is generated through charge transfer between the electrode and the ground. When a hand approaches the friction layer, the electric field is redistributed, causing negative charges to flow from the ground to the nanofiber electrode, producing a transient current in the external circuit. When the hand remains stationary, the system reaches an electrostatic equilibrium state. When the hand moves away from the friction layer, the negative charges flow back from the nanofiber electrode to the ground (Note S1). Based on the charge transfer effect, this principle enables the detection of approaching and departing movements. The proximity sensing layer is made from electrospun PVDF nanofibers, with the electrode part formed by magnetron sputtering technology to create interdigitated Ag electrodes on the nanofiber surface (Fig. S15A). To enhance surface charge density and charge capture ability, 2D MXene sheets were doped into PVDF. After electrospinning, a composite film with a spherical multiple physical network structure was prepared (Fig. S15B, C). This spherical multiple physical network exhibits excellent chemical stability and high specific surface area, effectively capturing and accumulating more negative charges. Additionally, the highly conductive Ti_3_C_2_T_x_ is uniformly distributed within the microspheres, increasing continuous conductive paths. The successful preparation of the proximity sensing layer film was confirmed by X-ray diffraction (XRD) (Fig. S16), and the interaction between PVDF and Ti_3_C_2_T_x_ sheets was studied. The (104) plane diffraction peak of Ti_3_C_2_T_x_ was observed at 36°, with the peak enhancement attributed to the bonding of H and F atoms on PVDF chains with surface functional groups of Ti_3_C_2_T_x_, ultimately forming polymer chains between Ti_3_C_2_T_x_ layers [59]. A similar trend was observed on the *β* (110/200) plane. When the Ti_3_C_2_T_x_ addition amount was too high, the diffraction peak width narrowed significantly, possibly due to reduced β-phase crystallinity in the composite film. To assess the effect of materials on non-contact sensing performance, we separately added MXene, PEDOT:PSS/MXene, and MXene/PEDOT:PSS/graphene to PVDF and tested the device performance (Fig. S17). Firstly, we made a preliminary attempt to mix PEDOT:PSS and MXene as sensing materials and conducted relevant experiments. However, the experimental results showed that this composite material did not improve the sensing performance significantly. On the contrary, the instability of the composite conductive network and the obstruction of the charge transfer path led to a decrease in the sensing performance, and the non-contact detection distance was about 1.2 m (lower than the sensor only with MXene, Fig. S17A). In addition, we attempted to introduce MXene/PEDOT:PSS/graphene into the sensing material and tested its performance. However, the test results showed that the output performance of the composite material was still degraded and the non-contact detection distance was about 1 m compared to MXene alone (Fig. S17B). The sensor only with MXene shows the highest performance i.e., non-contact detection distance of up to 2 m (Fig. 4H), which for the incorporated MXene nanosheets significantly enhances the friction charge density and thus the electrical output performance. Its high specific surface area and high conductivity enable the MXene nanosheets to capture and accumulate more negative charges while forming multiple conductive paths within the material, effectively improving the overall performance of the device. These experimental results suggest that the sensor only with MXene has the best performance and is selected in the work. To validate the performance of the proximity sensing layer, a series of experiments were designed, involving collision friction tests between the sensing layer and various materials using a linear motor **(**Fig. 4B**)**. The materials tested included nylon, mixed cellulose, knitted fabric, copper sheet, polyurethane, and thermoplastic polyurethane [60]. Due to significant differences in triboelectric effect intensity between different materials, different voltage values were generated for each material **(**Fig. 4C**)**. For example, the triboelectric effect between the nylon material and the sensing layer was significant, generating a high test voltage of 133 V. Figure 4D shows the test current for the interaction between the proximity sensing layer and nylon material, and Fig. 4E shows the test charge, which was 47 nC, indicating high charge transfer efficiency. The voltage test results for the interaction with nylon material are shown in Fig. 4F. To assess the impact of temperature changes on the sensing layer performance, voltage tests were conducted under different temperature conditions **(**Fig. 4G**)**. The sensing layer maintained high stability under various temperatures. To evaluate the effect of distance variations on the proximity sensing layer, signal changes at different distances from the nylon material were tested **(**Fig. 4H**)**. The voltage signal gradually decreased with increasing distance. Figure 4I shows the COMSOL simulation of the proximity sensing test, providing a visual representation of the electric field distribution and charge accumulation at different distances from the nylon material, further validating the working principle and performance characteristics of the sensing layer.

Experiments under different load conditions measured the peak power generated during the friction process **(**Fig. 4J**)**. The cycle stability of the proximity sensing layer was tested using collision-separation friction tests. After 4000 cycles (Fig. S18), the sensing layer maintained excellent stability. To verify the sensor's stable performance under different dynamic conditions, collision–separation friction tests were conducted at different frequencies. Figure 4K shows the voltage variations at different test frequencies, indicating that the sensing layer can output stable voltage signals under different test frequencies, demonstrating good frequency response characteristics.

### Multimodal Proximity–Pressure–Temperature Sensing Capability in Reality

**Fig. 5:**
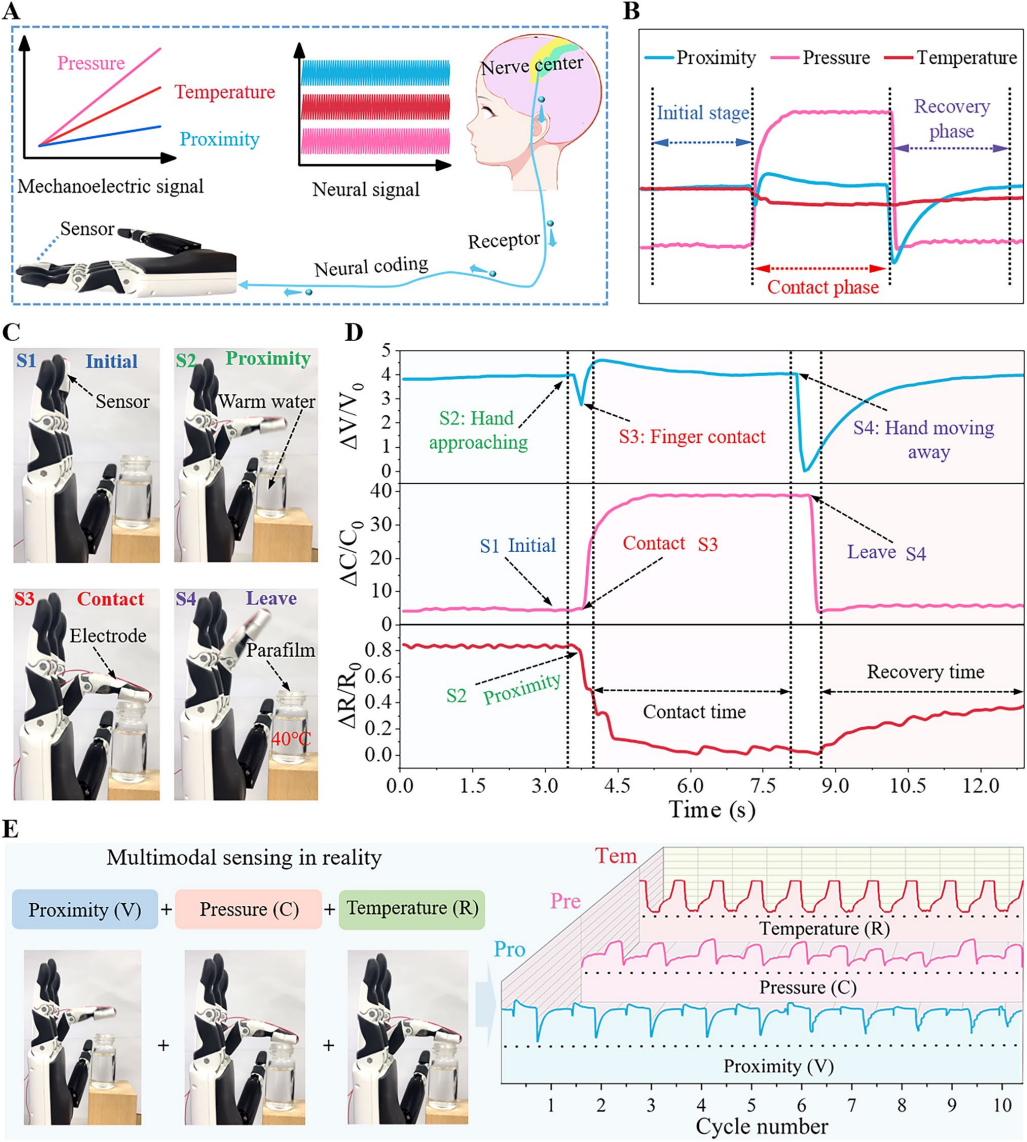
Simulation of the multimodal sensing capability by a robotic hand in a real-world scenario. **A** Schematic diagram illustrating the multimodal sensing process of robotics through mechanoelectrical signals mimicking human perception through neural signals. **B** Characteristic waveforms of these three sensing modes. **C** Simulation of four steps of the robotic hand touching a hot object, i.e., initial, approach, contact, and withdraw and **D** corresponding waveforms of these three sensing modes. **E** Combination of multimodal proximity–pressure–temperature sensing and tests by multiple cycles

Figure 5 explores the performance characteristics and working mechanisms of a robotic hand simulating real-world multimodal sensing. It also validates its excellent performance and broad application potential through a series of experiments and simulations. Multimodal sensing integrates mechanical, electrical, and neural signals, as illustrated in Fig. 5A. Figure 5B shows the characteristics of the three sensing modalities. Experimental measurements and analysis plotted the output characteristic curves for proximity, pressure, and temperature signals. The sensors were then attached to a robotic finger to simulate contact with a glass bottle containing warm water (to prevent humidity from affecting the sensors, the bottle's mouth was sealed with Parafilm), as shown in Fig. 5C. To more accurately evaluate the humidity effect on the performance of the sensor, we put the sensor in various environments with different humidities (40, 60, and 80% RH) and the performances were tested, as shown in Fig. S19. In the case of pressure sensing, humidity did not significantly affect the signal of pressure sensing (sensitivity variation less than 2%, Fig. S19A). For the proximity sensing layer, the voltage signals at different humidity levels were tested, and the results showed that the humidity had a small effect on the signals of the proximity sensing layer (variation less than 4.8%, Fig. S19B). As for the temperature signal, the humidity has little effect on the sensing ability (Fig. S19C). Thus, calibration of sensors before use can improve their reliability and detection accuracy. By controlling the motion trajectory of the robotic hand, we could accurately simulate the sensory process of a human finger from the initial approach to contact and then move away from the object (stages S1, S2, S3, and S4). Figure 5D illustrates the changes in sensor signals across the four stages, with detailed analysis and comparison. In stage S1, where the robotic finger remains stationary, the signals from all three sensors are stable. In stage S2, as the robotic hand begins to approach the warm water bottle, the proximity sensor starts to respond and generates a downward peak, while the pressure and temperature sensors remain stable. In stage S3, when the robotic hand makes contact with the warm water bottle, the pressure and temperature sensors begin to respond, with capacitance increasing and resistance decreasing. In stage S4, as the robotic hand moves away from the warm water bottle, all three sensors–proximity, pressure, and temperature-respond. The proximity sensor generates a downward peak and then gradually recovers, the pressure sensor capacitance decreases, and the temperature sensor resistance increases. Figure 5E shows the combined changes of proximity, pressure, and temperature signals. Here, the voltage change corresponds to proximity sensing, capacitance change corresponds to pressure sensing, and resistance change corresponds to temperature sensing. To verify the stability and reliability of the multimodal sensing system, multiple cyclic tests were conducted to measure variations in proximity, pressure, and temperature signals under different cycling conditions. The results demonstrate that all three signals exhibit good stability and consistency during multiple cycle tests, indicating that the multimodal sensing system has high reproducibility and reliability, capable of providing stable and reliable sensing data in practical applications.

### Intelligent Pillow Equipped with M-PPT for Human Sleep Monitoring

**Fig. 6:**
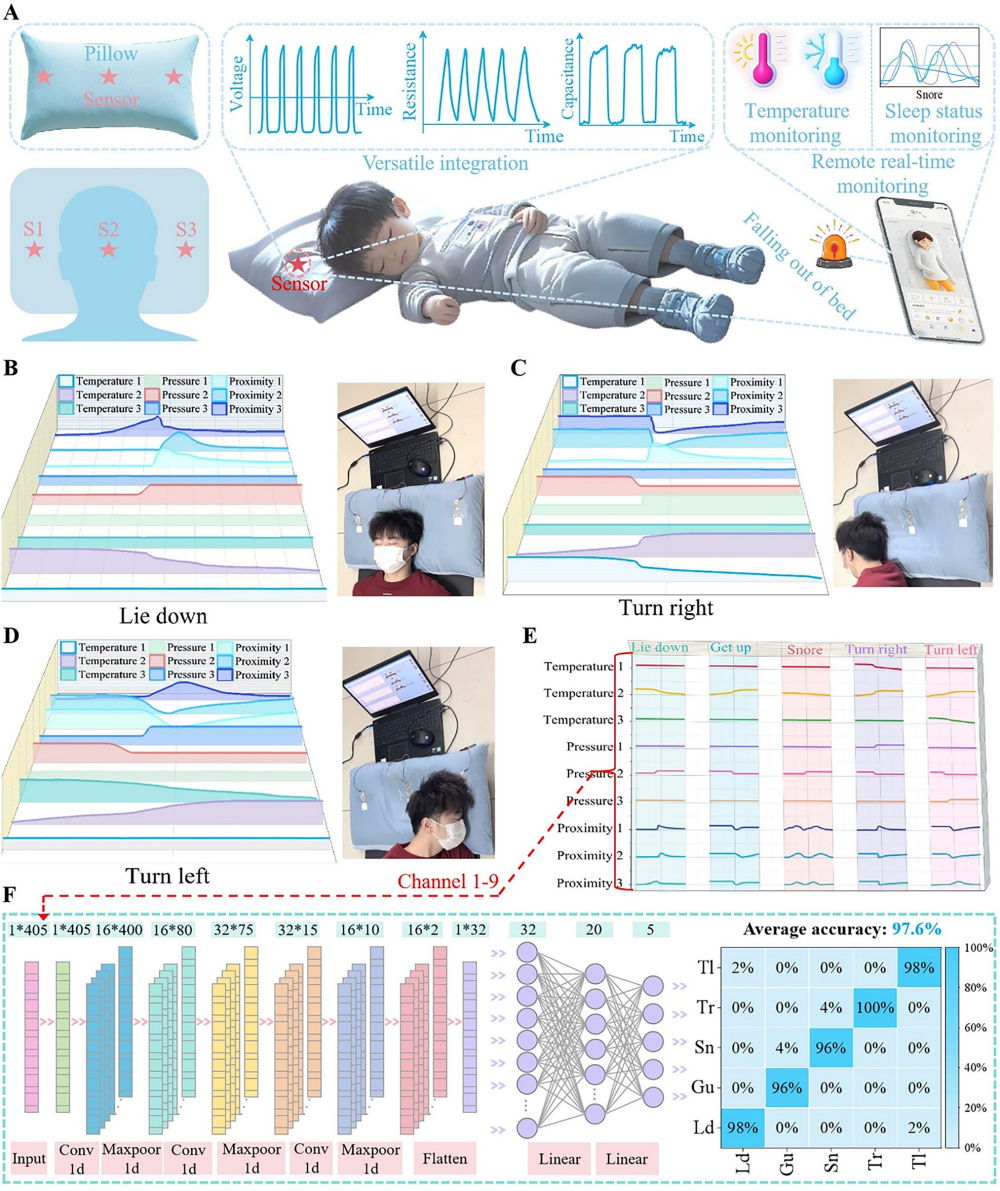
A multimodal sensor-integrated system applied to human sleep monitoring. **A** Three multimodal sensors are placed at different positions on the pillow to prevent potential health risks. **B** Signal response of multimodal sensors when the body lies down. **C** Signal response of multimodal sensors when the body turns to the right side. **D** Signal response of multimodal sensors when the body turns to the left side. **E** Sensor signal changes in five sleep states: lying down, getting up, snoring, turning to the right, and turning to the left. **F** Five different sleep states are trained using 1D CNN with a recognition success rate of 97.6%

Figure 6 illustrates the design, performance, and working mechanism of a multimodal sensing integrated system for human sleep monitoring and validates its excellent performance and broad application potential through a series of experiments and data analyses. Biosafety is a key guarantee for the application of the sensing patch in human sleep monitoring. Considering that the sensing patch needs to be in direct contact with the skin for a long time, we conducted comprehensive safety evaluation experiments on it, and the results showed that the sensing patch, as a safe and reliable biocompatible device, can be applied to human sleep monitoring without adverse health effects (Fig. S20). In detail, a comparative hemolysis test was performed as shown in Fig. S20A. Erythrocyte suspensions were prepared from fresh rat blood and positive control, negative control and experimental groups were set up. The experimental groups included nanofiber membrane (NFM) as well as hydrogel. The experimental results showed that the hemolysis rates of both experimental groups were low and met the biocompatibility requirements (Fig. S20A). Second, the hydrogel and nanofiber membrane (NFM) were attached to the back of the mice for 1 day, respectively. After the materials were removed, it was found that the mice's skin did not undergo redness or swelling, and there were no obvious changes (Fig. S20B). Then, the cytotoxicity testing was performed and the nanofiber film (NFM) as well as the hydrogel was evaluated in a mouse fibroblast cell line (L929). Figure S20C shows fluorescent live/dead double-stained images of L929 cells cultured on nanofiber membrane (NFM) as well as hydrogel after 24 and 48 h. And, L929 cells showed regular morphology after 24 and 48 h of incubation, indicating that the cells remained healthy even after prolonged incubation. To further assess the viability of L929 cells, the cell viability of the control and experimental groups at day the 1st day and the 2nd day was examined, as shown in Fig. S20D. The viability of L929 cells cultured on the nanofiber film as well as the hydrogel layer was sufficiently high to meet the evaluation criteria of no cytotoxic substances and demonstrated that the device had good biocompatibility. Thus, the sensing device prepared by us has good biocompatibility and will not harm human health. The multimodal sensing system integrates multiple sensors to precisely monitor and provide real-time feedback on different states during sleep [61]. Initially, three sensors are placed at different positions on a pillow **(**as shown in Fig. 6A**)**. To prove that multiple multimodal sensing patches are seamlessly integrated on different locations of a pillow, the images of the sensing patch on the pillow were taken, as shown in Fig. S21. It can be found that the interfacial connection between the patch and pillow is very smooth and tight, with no obvious gaps. The temperature sensor monitors nocturnal temperature variations, providing key information about temperature regulation (Fig. S22). Body surface temperature is an important parameter reflecting the physiological state of the body and sleep health and is closely related to sleep initiation, deep sleep maintenance, and sleep disorders. If the body surface temperature is too low or too high, it may lead to difficulty in falling asleep or shortened duration of deep sleep. In addition, different sleep stages are accompanied by small changes in body surface temperature, and by monitoring these changes, sleep structure and quality can be indirectly inferred. The proximity sensor helps analyze movement trajectories during sleep and whether there is frequent tossing and turning. The pressure sensor accurately records changes in head position, detecting different sleeping postures such as side-lying or supine, and identifying potentially problematic positions that could lead to poor sleep. By integrating this data, users can receive personalized sleep recommendations to improve sleep quality and prevent potential health risks. To demonstrate the capabilities of the multimodal sensing system in human sleep detection, we first examined the signal responses of the multimodal sensors when the volunteer was in a supine sleeping position, as shown in Fig. 6B. As the person lies down, the proximity sensor shows corresponding changes. After lying down, the sensor in the center of the pillow detects changes in contact pressure and body temperature and outputs corresponding signals. The sensors on the sides of the pillow respond only during the process of lying down, while pressure and temperature sensors remain stable. Figure 6C explores the signal responses of the multimodal sensors as the person turns to the right side from the supine position. As the person begins to turn right, moving the head away from the center of the pillow, the pressure and temperature signals from the central sensor start to recover, and the proximity sensor generates a corresponding peak. The side sensors show a response in proximity sensing while pressure and temperature sensing remain stable. Once the head is positioned on the right side, the sensor on the right side of the pillow detects changes in contact pressure and body temperature and outputs corresponding signals. Figure 6D examines the signal responses of the multimodal sensors as the person turns to the left side from the supine position. As the person starts to turn left, moving the head away from the pillow's center, the pressure and temperature signals from the central sensor begin to recover, and the proximity sensor generates a corresponding peak. The side sensors show a response in proximity sensing while pressure and temperature sensing remain stable. Once the head is positioned on the left side, the sensor on the left side of the pillow detects changes in contact pressure and body temperature and outputs corresponding signals. Combining signals from different turning directions provides a comprehensive analysis of changes in body posture during sleep. Figure 6E presents the sensor signal variations under five sleep states: lying down, getting up, snoring (as shown in Fig. S23), turning right, and turning left. Detailed data analysis plotted signal variation curves for different sleep states. These curves clearly show the signal features and changes for each sleep state. For instance, signal changes while lying down and getting up mainly reflect rapid changes in contact pressure and proximity sensing **(**Movie S1**)**, while right and left turning signal changes reflect the directional turning actions (shown by Movies S2 and S3). Snoring signals exhibit periodic vibration characteristics **(**Movie S4**)**. To further realize the recognition capabilities of the multimodal sensing system, a one-dimensional convolutional neural network (1D CNN) was used to train and test the five different sleep states, as shown in Fig. 6F (Ld: Lying down. Gu: Getting up. Sn: Snoring. Tr: Turning right. Tl: Turning left). The 1D CNN was built using the Pytorch framework, consisting of three convolutional units, one flattening layer, and two fully connected layers.

The convolutional units include 1D convolutional layers, 1D normalization layers, ReLU activation layers, and 1D max pooling layers for feature extraction. After convolutional units, the flattened layer expands all channel data into a 1D array of 36 neurons. The fully connected layers have 20 and 5 neurons, respectively. The data are processed through the 1D CNN, ultimately yielding five outputs representing the probability of each of the five sleep states. The state with the highest probability is identified as the current sleep state (Details of the training set, validation set, and test set for 1D CNN are given in Note S2). The model was trained over 5000 epochs, using cross-entropy loss to assess the difference between predicted and accurate values and gradient descent to update network weights. The final model achieved an accuracy of 97.6%. The multimodal sensing system, combined with advanced signal processing and machine learning algorithms, enables high-precision recognition and monitoring of complex sleep states, providing reliable technical support for personalized sleep quality analysis and health management.

## Conclusions

In this study, we developed a flexible multimodal proximity/pressure/temperature sensing patch (M-PPT) based on hydrogel. The hydrogel exhibits excellent mechanical properties, with strength and toughness remaining stable after multiple cycles, excellent water retention, and no weight loss after 8 days. The hydrogel's pressure sensing sensitivity is enhanced by the introduction of a microspherical structure design, with a pressure sensitivity of 30.6 kP^−1^ and a response time of only 5.6 ms, and a temperature sensing sensitivity of 0.5 °C^−1^ with good linearity. Non-contact sensing offers an ultra-wide detection range (2 m) and good durability. To verify the practical application of M-PPT, the ability of multimodal sensing was demonstrated by controlling the manipulator to touch the object to simulate a real scenario. In the end, multiple multimodal sensing patches are seamlessly integrated into different positions of the pillow, and the real-time information of temperature, pressure, and proximity signals are obtained from the interaction between human and pillow, and a 1D CNN is applied to analyze the multidimensional sensing data, successfully identifying five different sleep states. This innovation not only extends the application of hydrogels in wearable devices but also provides new insights for the design of future health monitoring systems.

## Supplementary Information

Below is the link to the electronic supplementary material.

Supplementary file1 (MP4 3031 kb)

Supplementary file2 (MP4 4261 kb)

Supplementary file3 (MP4 3977 kb)

Supplementary file4 (MP4 3447 kb)

Supplementary file5 (PDF 1633 kb)
