# Supplementary material for: A Flexible-Integrated Multimodal Hydrogel-Based Sensing Patch
Source: Nanomicro Lett. 2025 Feb 21;17:156. doi: 10.1007/s40820-025-01656-w (PMC11845634; doi:10.1007/s40820-025-01656-w)
Supplement: Supplementary file 5 — Supplementary file5 (PDF 1633 kb) [file 40820_2025_1656_MOESM5_ESM.pdf]

Supporting Information for

A Flexible Integrated Multimodal Hydrogel Based Sensing Patch

Peng Wang[2,//], Guoqing Wang[1,//], Guifen Sun[3], Chenchen Bao[1], Yang Li[1,4,6,*], Chuizhou Meng[3,*] and Zhao Yao[5,*]

[1] School of Information Science and Engineering, University of Jinan, Jinan 250022, P. R. China

[2] School of Mechanical Engineering, University of Jinan, Jinan 250022, P. R. China

[3] State Key Laboratory for Reliability and Intelligence of Electrical Equipment, Engineering Research Center of Ministry of Education for Intelligent Rehabilitation Device and Detection Technology, Hebei Key Laboratory of Smart Sensing and Human-Robot Interaction, School of Mechanical Engineering, Hebei University of Technology, Tianjin 300401, P. R. China

[4] School of Integrated Circuits, Shandong University, Jinan 250101, P. R. China

[5] College of Electronics and Information, Qingdao University, Qingdao 266071, P. R. China

[6] State Key Laboratory of Integrated Chips and Systems, Fudan University, Shanghai, 200433, P. R. China

[//] Peng Wang and Guoqing Wang contributed equally to this work.

*Corresponding authors. E-mail: yang.li@sdu.edu.cn (Yang Li); 2018108@hebut.edu.cn (Chuizhou Meng); yzh17@qdu.edu.cn (Zhao Yao)

Note S1 How the proximity sensing layer works in non-contact mode

The movement of the human body can induce spatial electrostatic induction with dynamic non-contact sensing characteristics. Initially, the human body and the frictional electronic skin are separated. Once the human body approaches the frictional electronic skin, the potential difference between the electrode layer and the ground induces electron flow, thereby generating a voltage signal. As the human body begins to move away from the frictional electronic skin, electrons flow in the opposite direction from the ground to the electrode layer, completing a full signal generation cycle.

Note S2 Training set, validation set and test set for 1DCNN

In terms of data preparation, we strictly divide the training set, validation set and test set to ensure the generalization ability and reliability of the model. The training set accounts for 70% of the data, which is used for the optimization of model weights; the validation set accounts for 15%, which is used for real-time assessment of the model's performance during the training process to prevent overfitting; and the test set accounts for 15%, which is used for the final assessment of the model's practical application effect. After the data is processed by 1D CNN, five values are finally output, representing the probability of each of the five sleep states, and the state with the highest probability is determined as the current sleep state.

Note S3 Rapid temperature recovery mechanism

In our temperature sensing system, carriers in the hydrogel play a key role in the temperature change process. Specifically, ion transport in hydrogels is a thermally activated process, i.e., as the temperature increases, the ion transport rate increases rapidly, which leads to a rapid decrease in the resistance of the hydrogel. Correspondingly, when the temperature decreases,

the ion transport rate decreases rapidly, resulting in a rapid increase in the resistance of the hydrogel. The increase and decrease in temperature also triggers a microscopic phase transition within the hydrogel. This phase change process helps to improve the response and recovery speed of the temperature sensor. When the temperature changes, the microstructure within the hydrogel adapts to the temperature difference and responds quickly or recovers quickly.

Supplementary Figures and Tables

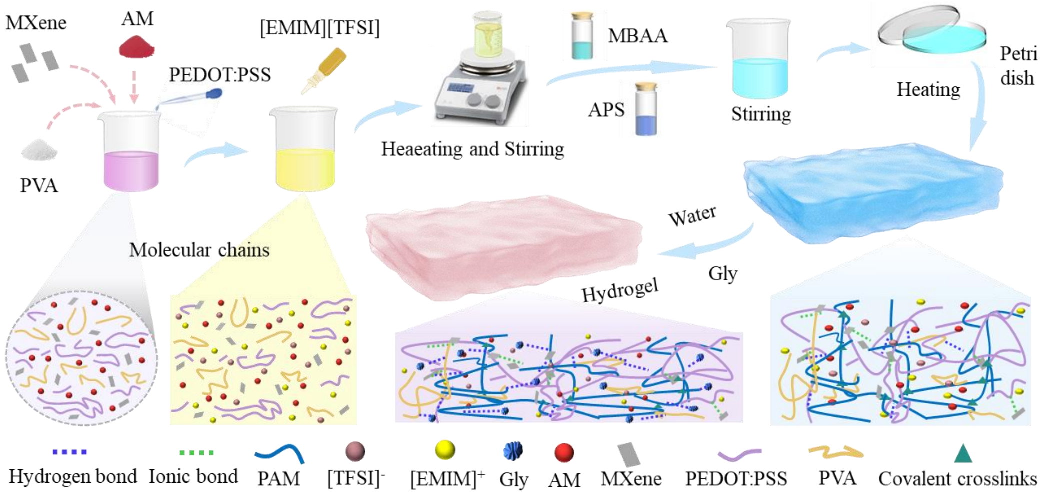

Fig. S1 Flow chart for the preparation of hydrogel-based pressure-temperature sensors

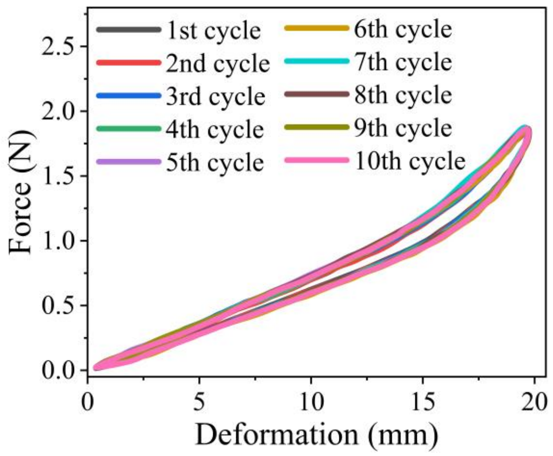

Fig. S2 Stress-strain curves of hydrogels

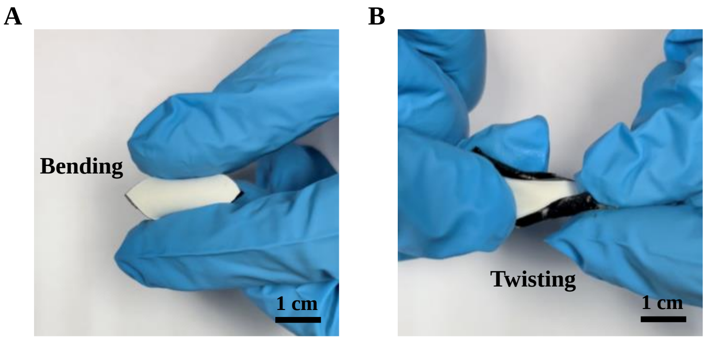

Fig. S3 (**A**) Bending test for sensing patches. (**B**) Twisting test on sensing patches

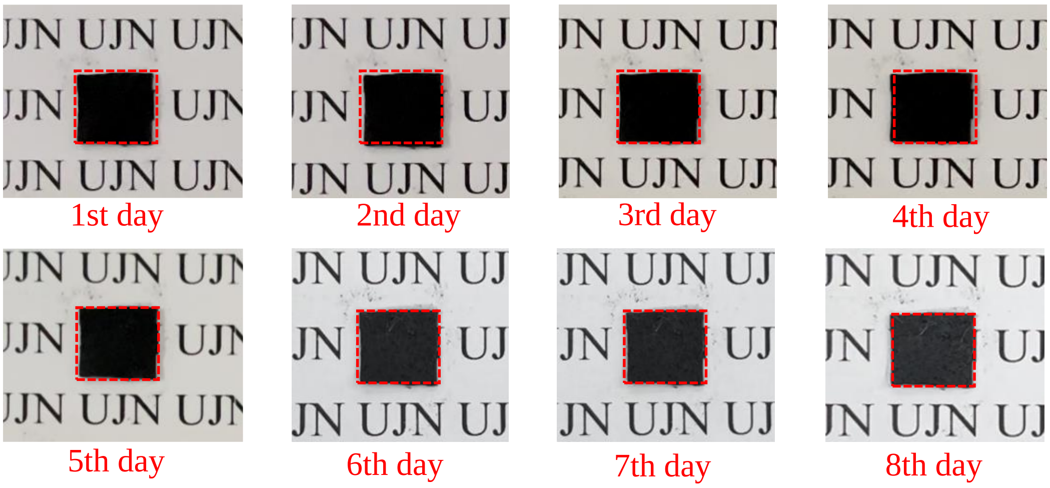

Fig. S4 Water retention test of hydrogels over eight days

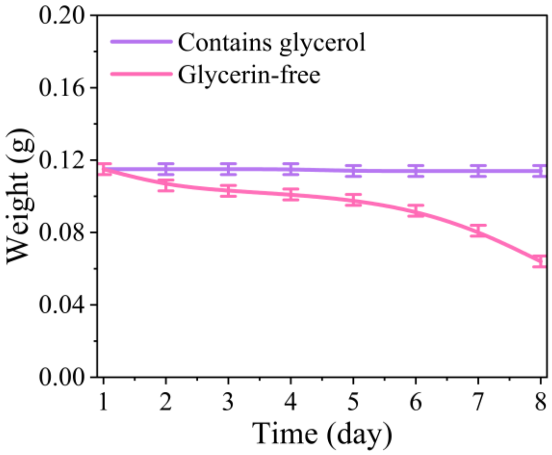

Fig. S5 Weight change over eight days for glycerin-containing and non-glycerin-free hydrogels

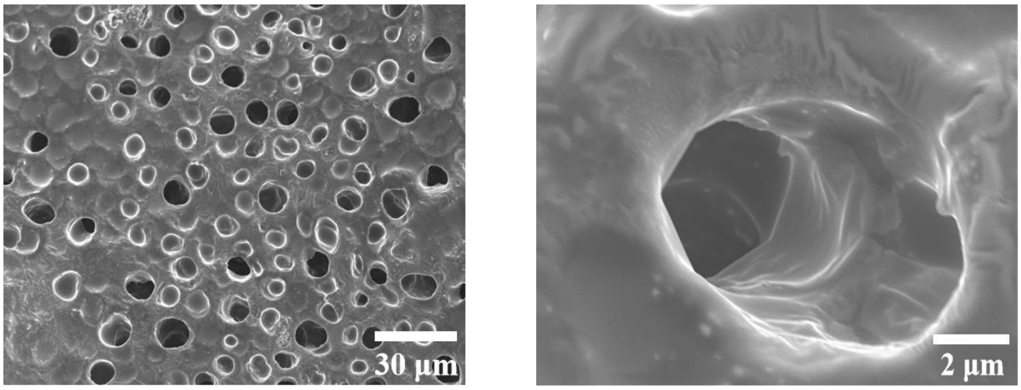

Fig. S6 After freeze-drying, the porous structure inside the hydrogel

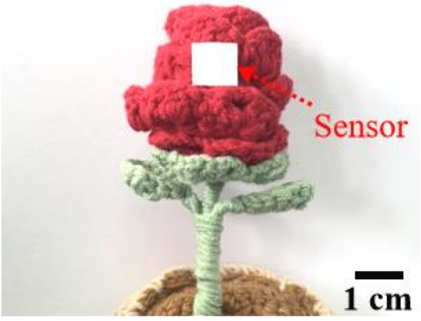

Fig. S7 Preparation of hydrophobic layer TPU film with micro-cone structures

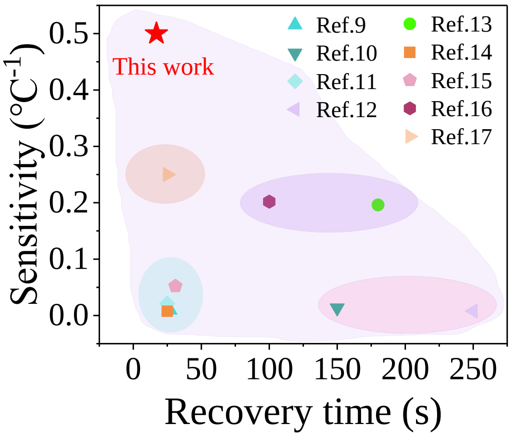

Fig. S8 Performance comparison of the sensor with previously reported works [S9-S17]

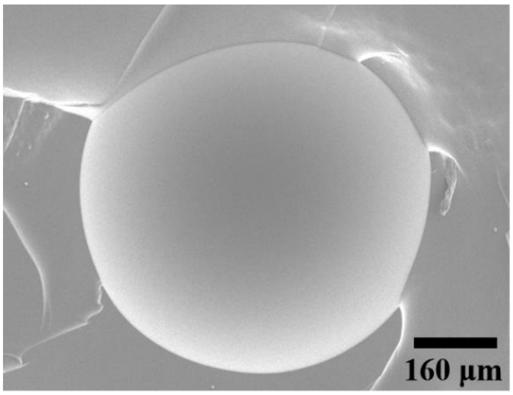

Fig. S9 SEM image characterization of spherical microstructures on the surface of hydrogels

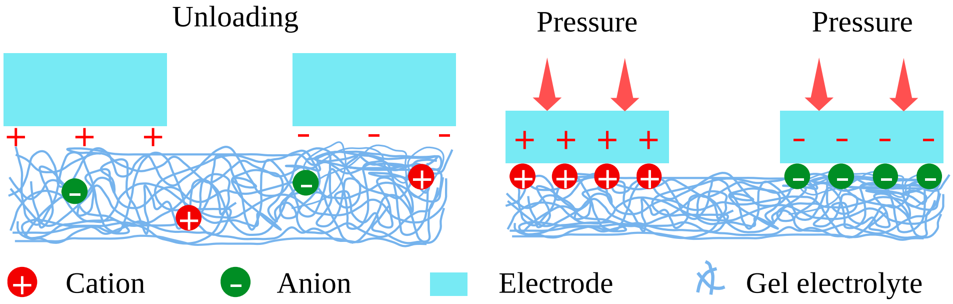

Fig. S10 Schematic diagram of the working principle of a hydrogel-based pressure sensor

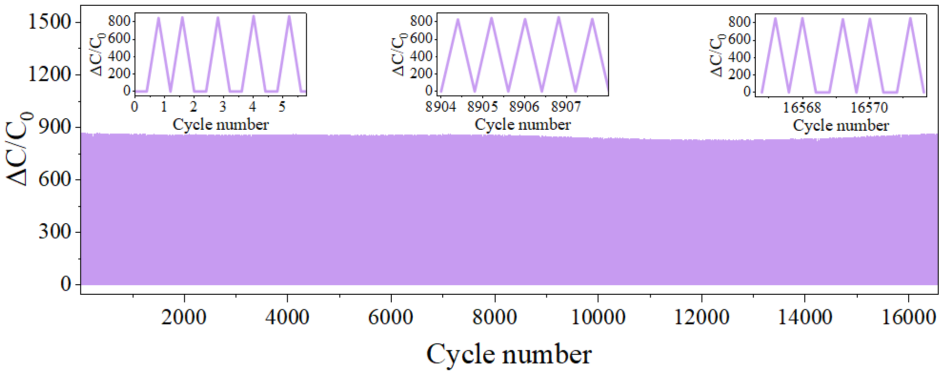

Fig. S11 Hydrogel-based pressure sensor ~16500 cycles stability test

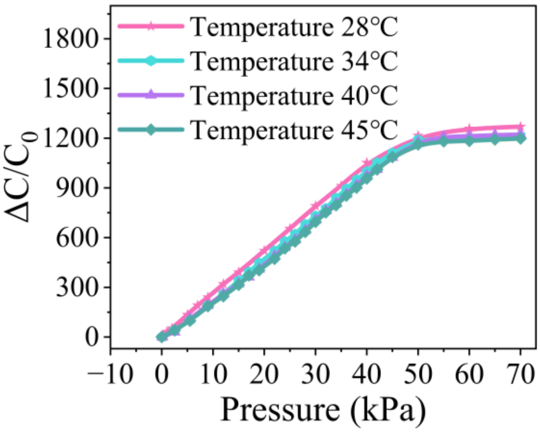

Fig. S12 Stable performance of the pressure sensor at different temperatures

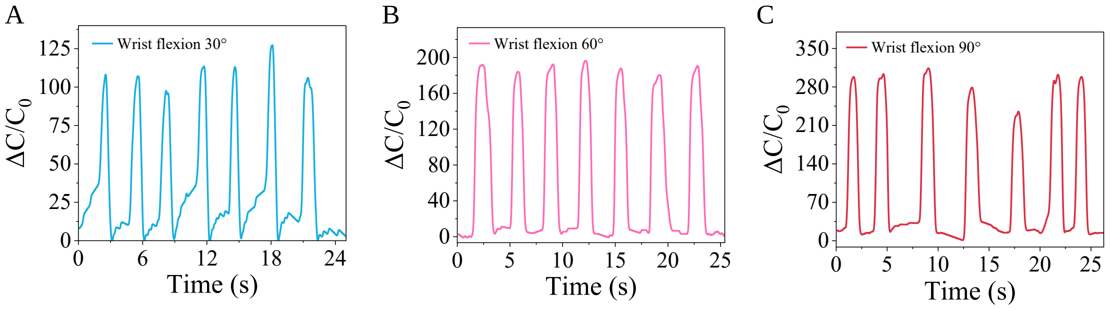

Fig. S13 Hydrogel-based pressure sensors are used to detect pressure changes at different angles of bending of the wrist. (**A**) Signal change at 30° wrist flexion. (**B**) Signal change at 60° wrist flexion. (**C**) Signal change at 90° wrist flexion

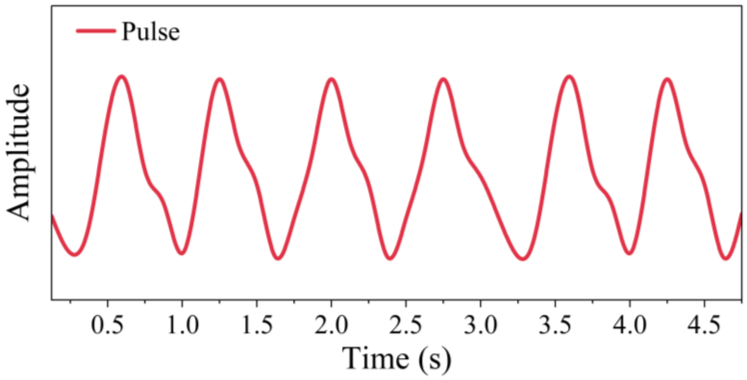

Fig. S14 The process of downward transfer when a 0.5 ml drop of water falls on the hydrophobic side

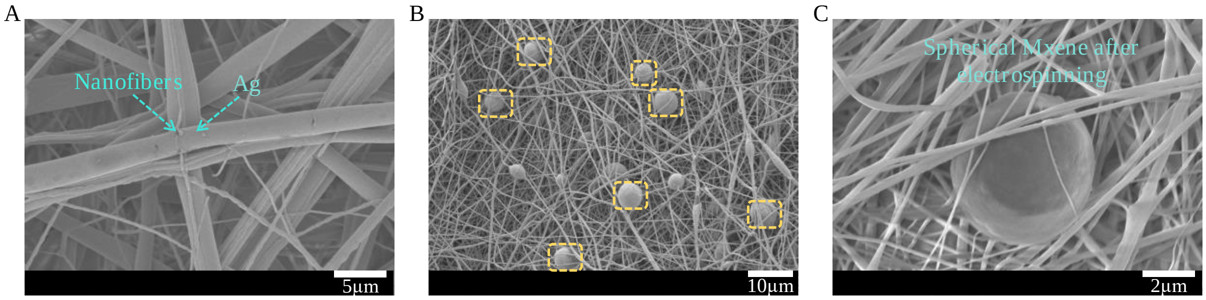

Fig. S15 SEM characterization of the microstructure of the prepared proximity sensing layer. (**A**) SEM image of the Ag/nanofiber electrode layer. (**B-C**) SEM images of the spherical MXene on the surface of the proximity sensing layer

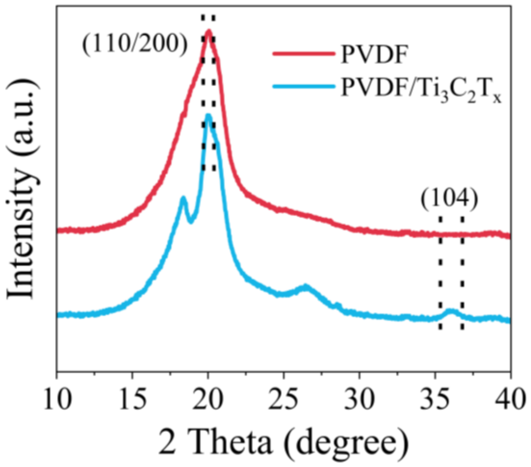

Fig. S16 XRD analysis of proximity sensing layers

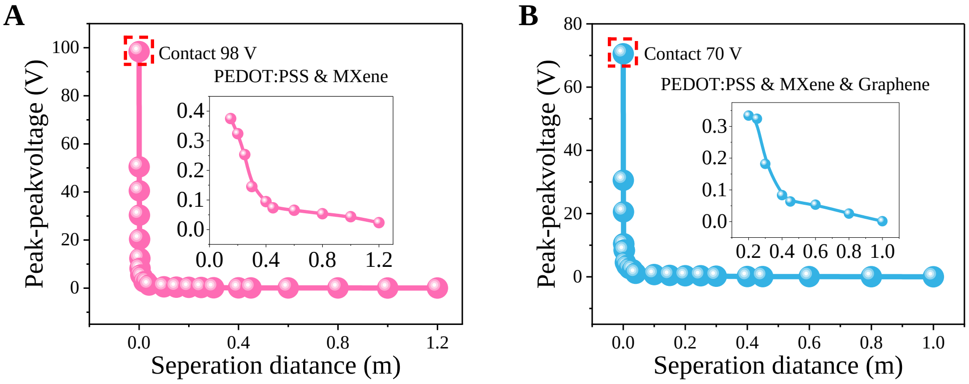

Fig. S17 (**A**) PEDOT:PSS mixed with MXene non-contact detection distance of 1.2m. (**B**) PEDOT:PSS mixed with MXene and Graphene with a non-contact detection distance of 1 m

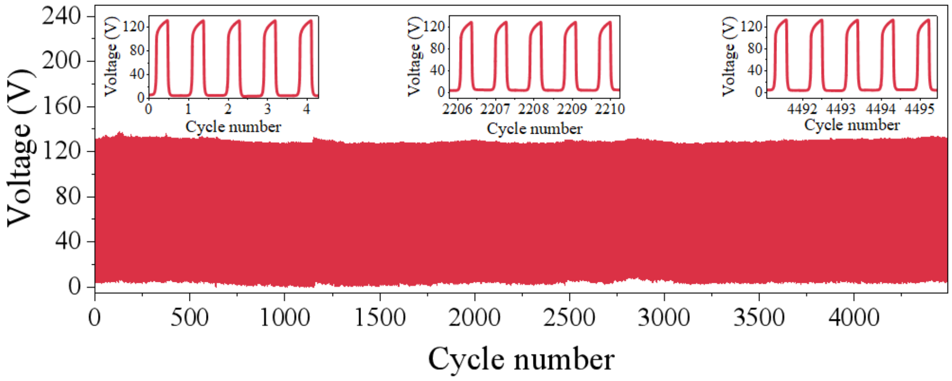

Fig. S18 Cyclic stability of the proximity sensing layer after 4000 crash-separation friction tests

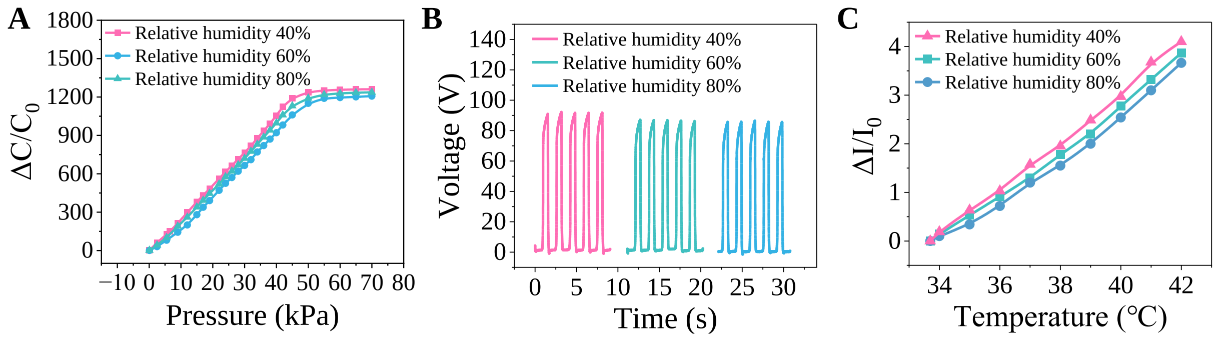

Fig. S19 (**A**) Effect of humidity on the sensitivity of pressure sensors. (**B**) Effect of humidity on the voltage signal of the proximity sensing layer. (**C**) Effect of humidity on the sensitivity of the temperature sensing layer

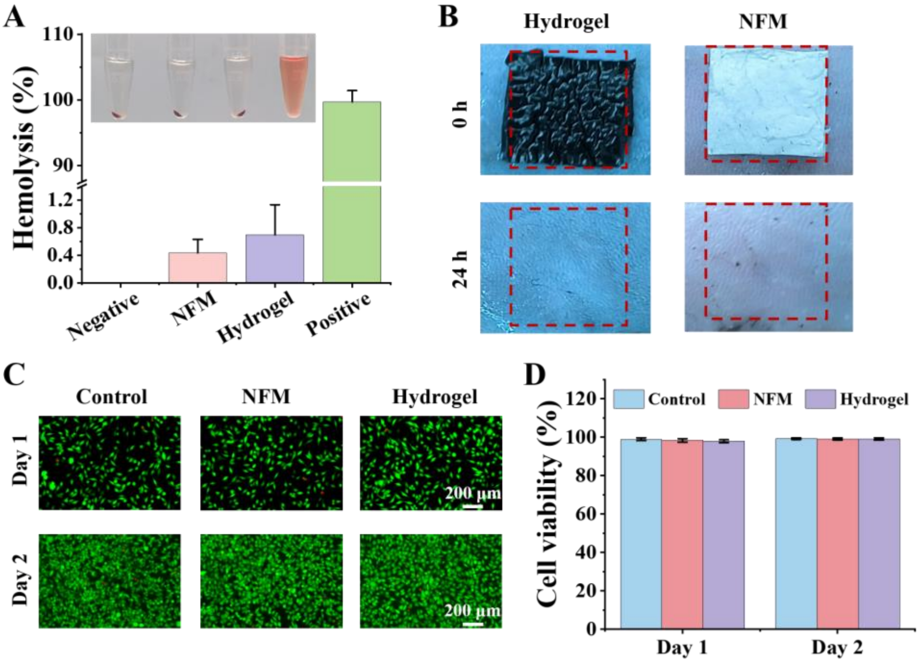

Fig. S20 (**A**) Hemolysis rate test. (**B**) Mouse skin red and swollen test. (**C**) Cytotoxicity test. (**D**) Cell viability test

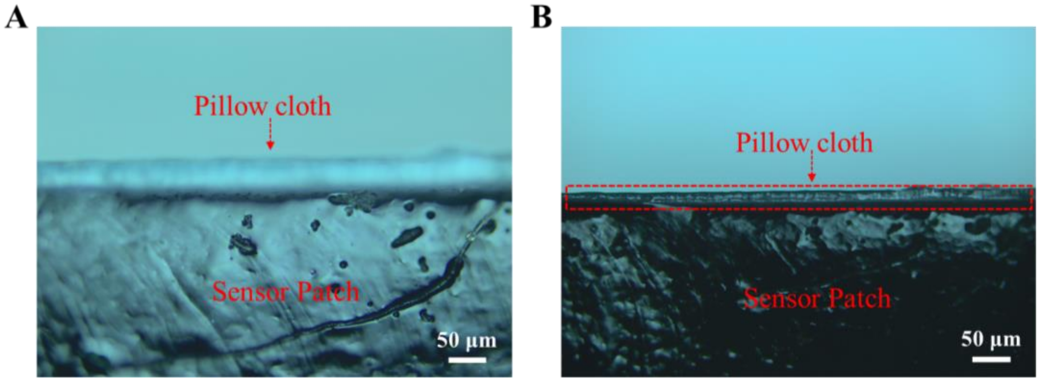

Fig. S21 Optical microscopy image of the "seamless" interface between the sensing patch and the pillow cloth

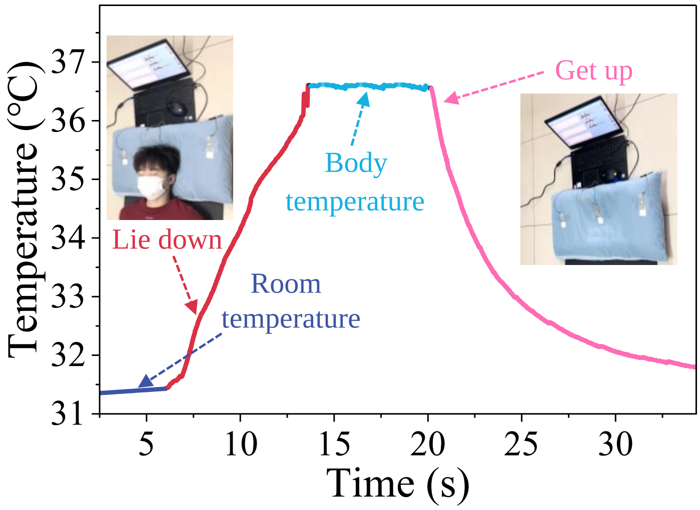

Fig. S22 The human sleep monitoring system detects the temperature of the human body in different states of lying down and getting up

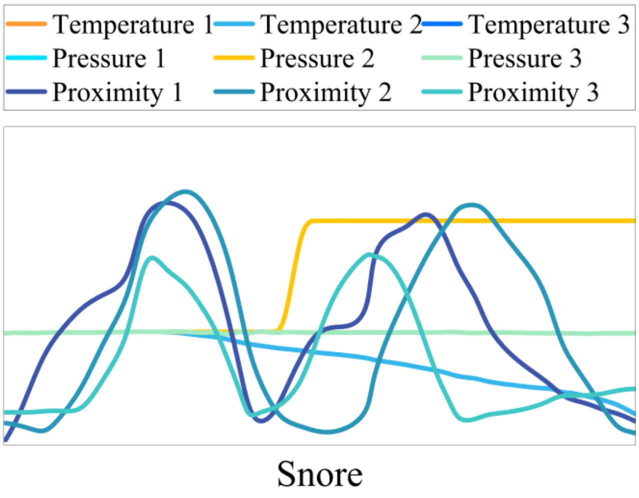

Fig. S23 The human sleep monitoring system is able to accurately capture and record changes in signals when snoring

Table S1 Comparison of performance of capacitive sensors based on different nanomaterials

Sensing material	Sensitivity (kPa^{-1})	Maximum detection pressure (kPa)	Response time (ms)	Stability	Refs.
CNTs/Pure ethanol/Ecoflex	2.13	3	100	100	[S1]
AgNWs/PDMS	0.831	10	30	10000	[S2]
PVA/PDMS/Salt	0.18	50	52	1000	[S3]
PDMS/PVDF/$BaTiO_3$	5	50	25	10000	[S4]
AgNWs/graphene	1.9	20	100	1000	[S5]
Ecoflex/PEN/ITO	1.277	0.4	100	10000	[S6]
PVA/KOH/KI/GL	0.3199	65	80	1400	[S7]
PVA/H_3PO_4	20.98	37.5	30	6000	[S8]
AM/MXene/PEDOT：PSS/PVA/[EMIM][TFSI]	30.6	70	5.6	16500	This work

Supplementary References

[S1] F. Liu, S. Dai, J. Cao, Z. Zhang, G. Cheng et al., CNTs based capacitive stretchable pressure sensor with stable performance. Sens. Actuat. A Phys. **343**, 113672 (2022). https://doi.org/10.1016/j.sna.2022.113672

[S2] R. Shi, Z. Lou, S. Chen, G. Shen, Flexible and transparent capacitive pressure sensor with patterned microstructured composite rubber dielectric for wearable touch keyboard application. Sci. China Mater. **61**, 1587–1595 (2018). https://doi.org/10.1007/s40843-018-9267-3

[S3] H. Cui, Y. Liu, R. Tang, J. Ren, L. Yao et al., A sensitive and flexible capacitive pressure sensor based on a porous hollow hemisphere dielectric layer. Micromachines **14**, 662 (2023). https://doi.org/10.3390/mi14030662

[S4] C.-R. Yang, M.-F. Lin, C.-K. Huang, W.-C. Huang, S.-F. Tseng et al., Highly sensitive and wearable capacitive pressure sensors based on PVDF/$BaTiO_3$ composite fibers on PDMS microcylindrical structures. Measurement **202**, 111817 (2022). https://doi.org/10.1016/j.measurement.2022.111817

[S5] F. Liu, F. Han, L. Ling, J. Li, S. Zhao et al., An omni-healable and highly sensitive capacitive pressure sensor with microarray structure. Chemistry **24**, 16823–16832(2018). https://doi.org/10.1002/chem.201803369

[S6] S.-W. Kim, G.-Y. Oh, K.-I. Lee, Y.-J. Yang, J.-B. Ko et al., A highly sensitive and flexible capacitive pressure sensor based on alignment airgap dielectric. Sensors **22**, 7390 (2022). https://doi.org/10.3390/s22197390

[S7] Y. Zeng, Y. Qin, Y. Yang, X. Lu, A low-cost flexible capacitive pressure sensor for health detection. IEEE Sens. J. **22**, 7665–7673 (2022). https://doi.org/10.1109/JSEN.2022.3158354

[S8] Y. Chen, Y. Qin, X. Zhang, A. Zheng, Q. Xia, Hierarchical *Arete* architecture-enabled iontronic pressure sensor with high linearity and sensitivity. Adv. Mater. Technol. **7**, 2200322 (2022). https://doi.org/10.1002/admt.202200322

[S9] F. Li, H. Xue, X. Lin, H. Zhao, T. Zhang, Wearable temperature sensor with high resolution for skin temperature monitoring. ACS Appl. Mater. Interfaces **14**, 43844–43852 (2022). https://doi.org/10.1021/acsami.2c15687

[S10] T.Q. Trung, S. Ramasundaram, B.U. Hwang, N.E. Lee, An all-elastomeric transparent and stretchable temperature sensor for body-attachable wearable electronics. Adv. Mater. **28**, 502–509 (2016). https://doi.org/10.1002/adma.201504441

[S11] C. Yan, J. Wang, P.S. Lee, Stretchable graphene thermistor with tunable thermal index. ACS Nano **9**, 2130–2137 (2015). https://doi.org/10.1021/nn507441c

[S12] H. Yang, D. Qi, Z. Liu, B.K. Chandran, T. Wang et al., Soft thermal sensor with mechanical adaptability. Adv. Mater. **28**, 9175–9181 (2016). https://doi.org/10.1002/adma.201602994

[S13] J. Wu, Z. Wu, Y. Wei, H. Ding, W. Huang et al., Ultrasensitive and stretchable temperature sensors based on thermally stable and self-healing organohydrogels. ACS Appl. Mater. Interfaces **12**, 19069–19079 (2020). https://doi.org/10.1021/acsami.0c04359

[S14] Y. Ouyang, X. Wang, X. Liu, M. Hou, M. Zheng et al., Spider-web and ant-tentacle doubly bio-inspired multifunctional self-powered electronic skin with hierarchical nanostructure. Adv. Sci. **8**, e2004377 (2021). https://doi.org/10.1002/advs.202004377

[S15] L. Chen, X. Yao, X. Liu, Z. Luo, C. Ye et al., High throughput In–situ temperature sensor array with high sensitivity and excellent linearity for wireless body temperature monitoring. Small Struct. **3**, 2200080 (2022). https://doi.org/10.1002/sstr.202200080

[S16] Y. Lu, H. Zhang, Y. Zhao, H. Liu, Z. Nie et al., Robust fiber-shaped flexible temperature sensors for safety monitoring with ultrahigh sensitivity. Adv. Mater. **36**, e2310613 (2024). https://doi.org/10.1002/adma.202310613

[S17] F. Chen, H. Deng, G. Li, X. Li, J. Pan et al., High-sensitivity wearable multi-signal sensor based on self-powered MXene hydrogels. Chem. Eng. J. **489**, 151221 (2024). https://doi.org/10.1016/j.cej.2024.151221
